# Oslo government district bombing and Utøya island shooting July 22, 2011: The immediate prehospital emergency medical service response

**DOI:** 10.1186/1757-7241-20-3

**Published:** 2012-01-26

**Authors:** Stephen JM Sollid, Rune Rimstad, Marius Rehn, Anders R Nakstad, Ann-Elin Tomlinson, Terje Strand, Hans Julius Heimdal, Jan Erik Nilsen, Mårten Sandberg

**Affiliations:** 1 Air Ambulance Department, Oslo University Hospital, Oslo, Norway; 2 Department of Research, Norwegian Air Ambulance Foundation, Drøbak, Norway; 3 University of Bergen, Bergen, Norway; 4 Department of Anaesthesia and Intensive Care, Oslo University Hospital, Oslo, Norway; 5 Ambulance Department, Oslo University Hospital, Oslo, Norway; 6 Emergency Department, Oslo University Hospital, Oslo, Norway; 7 Department of Prehospital Medicine, Vestre Viken Health Enterprise, Drammen, Norway; 8 National Centre for Prehospital Emergency Care, Oslo, Norway; 9 University of Oslo, Oslo, Norway

**Keywords:** Terrorism, Mass Casualty Incidents, Triage, Prehospital Emergency Care

## Abstract

**Background:**

On July 22, 2011, a single perpetrator killed 77 people in a car bomb attack and a shooting spree incident in Norway. This article describes the emergency medical service (EMS) response elicited by the two incidents.

**Methods:**

A retrospective and observational study was conducted based on data from the EMS systems involved and the public domain. The study was approved by the Data Protection Official and was defined as a quality improvement project.

**Results:**

We describe the timeline and logistics of the EMS response, focusing on alarm, dispatch, initial response, triage and evacuation. The scenes in the Oslo government district and at Utøya island are described separately.

**Conclusions:**

Many EMS units were activated and effectively used despite the occurrence of two geographically separate incidents within a short time frame. Important lessons were learned regarding triage and evacuation, patient flow and communication, the use of and need for emergency equipment and the coordination of helicopter EMS.

## Background

On July 22, 2011, Norway was struck by two terrorist attacks. In the first attack, a car bomb exploded in the Oslo government district. The bomb comprised an ammonium nitrate/fuel oil (ANFO) mixture or "fertiliser bomb". Eight people were killed in the explosion. Two hours later, a lone gunman attacked a political youth camp on Utøya island, approximately 40 kilometres from Oslo, and killed 69 civilians. A single perpetrator carried out both attacks.

The scale of the July 22, 2011 attacks and the resulting emergency medical service (EMS) response was unprecedented in Norway. The massive EMS response crossed jurisdictional lines and involved responders from multiple agencies throughout the region. In this paper, we describe the immediate prehospital EMS response to the July 22, 2011 attacks.

## Methods

### The Norwegian EMS

The backbone of the Norwegian EMS is provided by on-call general practitioners (GPs) and ground ambulances [1]. According to national regulations, all ambulance units must be staffed by at least one certified emergency medical technician (EMT) [2]. However, most units are staffed by two EMTs, and in most urban systems, at least one EMT is a trained paramedic. The ambulance service is government-funded and organised under local health enterprises. In Oslo, a physician-manned ambulance is operational during the daytime on weekdays and is staffed by certified or in-training anaesthesiologists.

Since 1988, a national government-funded air ambulance system has provided rapid access to advanced life support by specially trained anaesthesiologists [3,4]. This service consists of 11 helicopter EMS (HEMS) bases and 7 fixed-wing EMS bases, all operating 24 hours a day [5]. All HEMS units are staffed by an anaesthesiologist and a HEMS paramedic. Six search-and-rescue (SAR) helicopter bases operated by the Royal Norwegian Air Force under the jurisdiction of the Ministry of Justice and the Police are also an integral part of the national air ambulance system [1]. These helicopters are also staffed by an anaesthesiologist and a rescue-man [5]. As back-up during non-flying weather conditions or for incidents close to the helicopter base, all civilian and some SAR helicopter bases use rapid response cars [6].

Twenty emergency medical communication centres (EMCC) coordinate EMS resources and on-call GPs in their region. Nurses who answer public emergency calls through the national toll-free medical emergency number (113) staff the EMCCs together with EMT-trained operators who coordinate the EMS and HEMS resources in the region.

### The Norwegian trauma care system

Norway has a three-tiered system of local, central and university hospitals. The catchment areas for the local and central hospitals range from 13,000 to 400,000 people. University hospitals serve as trauma referral centres and provide definitive care for populations ranging from 460,000 to 2.5 million [7].

### EMS major incident preparedness

A standard for major incident triage does not exist in Norway; most triage systems are confined to local systems [8]. However, a framework for the management, organisation and coordination of major incident scenes has been established [9]. According to this framework, incident command is managed by a police officer. Other branches involved are represented by their respective branch scene commanders, and the most central are those from the fire and rescue and EMS. An ambulance scene commander (ASC) is responsible for coordinating all on-scene EMS resources, and a medical scene commander (MSC) is the leading medical person on scene, who is responsible for triage and on-scene medical treatment. In addition, the scene is organised with parking and loading points for EMS vehicles and casualty-clearing stations.

A light emergency stretcher system (LESS), developed in the Optimal Patient Evacuation Norway (OPEN) concept, is available in several EMS and SAR systems in Norway [10]. These stretchers are stored in transport-friendly bags of five and are insulating and radiolucent. Within the intended function for which they were developed [10], they are intended to follow the patient from first contact to hospital arrival, thus avoiding unnecessary patient manipulation.

### Scene descriptions and EMS resources; Oslo

Oslo is the capital of Norway and has a population of approximately 605,000 inhabitants. The immediate urban area around Oslo, however, accounts for nearly one million people. The Oslo government district is located in the business district of Oslo and consists of several buildings housing most of the ministries. Traditionally, the area has been open to the public, and all nearby streets have been accessible to civilian vehicles.

The road transport time from the bomb site to Oslo University Hospital (OUH) takes 5-10 minutes. OUH is the major health institution in Oslo and consists of three university hospital campuses: Rikshospitalet, Ullevål and Aker (Table 1). OUH-Ullevål (OUH-U) is a combined primary and regional referral trauma centre that serves almost half the Norwegian population. A combined casualty clinic and GP-staffed primary health care facility in the Oslo business district attends to walk-in patients and is located 2-3 minutes away from the government district by vehicle. The ambulance department of OUH has 15 ambulance stations and 43 ambulance units (25 units on-call day and night) in Oslo and the surrounding municipalities. In addition, an ambulance commander is on duty day and night in a separate vehicle and acts as the ASC in incidents involving multiple units. The air ambulance base of OUH with two HEMS units is located in Lørenskog, which is just outside the city limits of Oslo. The EMCC of Oslo and Akershus coordinate the activity of all the EMS resources of OUH.

**Table 1:** The distance by road from the scenes of July 22, 2011 to the Oslo University Hospital campuses and the hospitals of the Vestre Viken Health Enterprise.

Hospital		Distance (km)*	
*Name*	*Type*	*Utøya island*	*Oslo^2^*

Oslo University Hospital			

Rikshospitalet	University	34	5
Ullevål	University^1^	38	4
Aker	University	44	5.5
Casualty clinic	Local	40	1.5

Vestre Viken Health Enterprise			

Ringerike	Local	16	n/a
Drammen	Central	43	n/a
Asker-Bærum	Local	20	n/a
Kongsberg	Local	90	n/a

^1^Dedicated trauma hospital; ^2^Bomb incident site

*Distances are approximate because there are several alternate routes

n/a = Not applicable because no patients were transported to this hospital from the site

### Scene description and local EMS resources; Utøya

**Figure 1:**
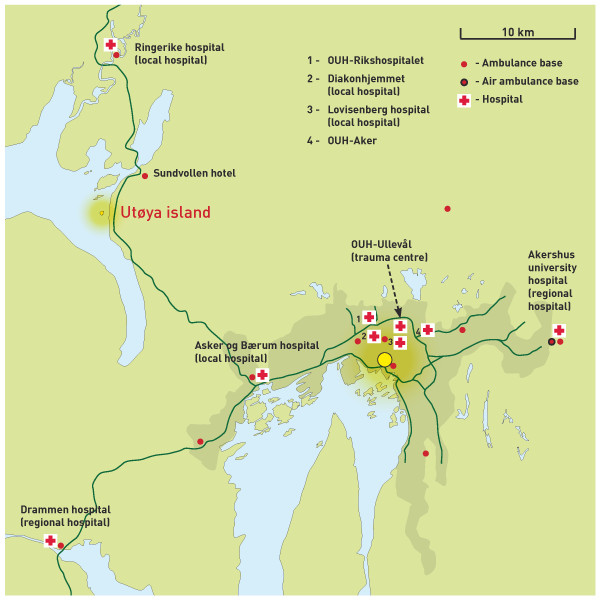
**Map of the greater Oslo area including Utøya depicting all (H)EMS bases in the area, the hospitals and main roads**.

Utøya island is 39 kilometres from central Oslo and lies in the Tyrifjorden lake (Figure 1). The 0.12 square kilometre island is owned by the youth organisation of the Norwegian Labour Party and is known for its annual summer camp. The island can only be reached by boat from the mainland. A small ferry that can accommodate one car is the only organised transport route to the island. The shortest distance from Utøya to the mainland is approximately 630 metres.

The Vestre Viken Health Enterprise (VVE) is responsible for the specialist health services and the EMS in the region. The regional hospital resources and their distance to Utøya island are described in Table 1 and depicted in Figure 1. The ambulance service in VVE has 17 ambulance stations with 24 ambulances operating day and night and an additional 5 daytime ambulances. The HEMS base at Ål covers the VVE region together with the HEMS units from OUH. The EMCC of Buskerud coordinates the activity of all EMS resources of VVE.

### Environmental conditions

July 22, 2011 was a Friday during the Norwegian general staff vacation period. In Oslo, the midday weather was overcast with some light rain and a moderate north-northeast breeze. The air temperature was between 15 and 17°C. The weather conditions in the Utøya island area were similar, with light rain throughout the afternoon. The air temperature was between 14 and 15°C in the area, and the water temperature in the Tyrifjorden lake was 14°C.

### Study design

This is a retrospective observational study of available and relevant anonymous data on (H)EMS activity during the first 24 hours following the attacks of July 22, 2011. The CONsensus Guidelines on Reports of Field Interventions in Disasters and Emergencies (CONFIDE) was used in the drafting of this epidemiological assessment [11]. The heads of the involved prehospital services, the research directors of both institutions (OUH and VVE) and the Data Protection Official approved the data collection from relevant sources within OUH and VVE. Because the Data Protection Official and the research directors approved the study as a quality improvement project, formal approval from the Regional Committee for Medical and Health Research Ethics was considered to be unnecessary.

### Data sources and variables

The following data sources were screened for system and patient characteristic descriptors as well as process mapping variables [12] related to the prehospital EMS in the first 24 hours following the bombing in Oslo and the shootings at Utøya island:

• Communication log, Acute Medical Information System (AMIS) (Nirvaco AS, Oslo, Norway) of the EMCCs in Oslo, Akershus and Buskerud

• EMS operational data from OUH and VVE

• Flight log of Norwegian Air Ambulance

• Written reports from the prehospital EMS of OUH and VVE

• Written reports from the OUH casualty clinic and the OUH emergency department

• Data from the public domain regarding the incidents in Oslo and at Utøya island and the prehospital EMS activity

All points of time are reported in Central European Summer Time: GMT +2 (local time). Where a specific time was not recorded in any records, the time was estimated, and these times are reported as approximate.

## Results

### The Oslo government district scene

Table 2 shows an overview of the events related to the EMS response following the bomb attack on the Oslo government district with point of time.

**Table 2:** Timeline of the EMS response to the Oslo government district bombing.

Event	Time
Oslo government district bomb detonates	15:25

Oslo EMCC receives first calls from the public regarding the bomb	15:26

First ambulance unit arrives on scene	15:28

First victim arrives on foot at Oslo casualty clinic	15:33

Ambulance Scene Commander declares a major incident	15:33

Civilian bus requisitioned by EMS at the bombsite	15:35

Both OUH-HEMS crews dispatched	15:40

First victim arrives at Oslo University Hospital Ullevål	15:51

Forty-one ambulance units available at casualty-clearing station 2	15:51

Seventh victim arrives at Oslo University Hospital Ullevål	16:10

One OUH-HEMS crew commissioned for SAR and triage in one of the bombed government buildings	16:40

Decommissioning of units from Oslo scene initiated	17:00

EMCC = Emergency Medical Communication Centre, EMS = Emergency Medical Service, OUH-HEMS = Helicopter Emergency Medical Service of Oslo University Hospital, SAR = Search and Rescue

### Alarm, dispatch and initial response

The bomb in the Oslo government district detonated at 15:25. Within one minute, the Oslo EMCC received the first call from the public regarding the explosion. Twelve ambulance units in the area were dispatched and arrived on scene within minutes. Among the first arriving units was the ambulance commander, who assumed the role of ASC, and the physician-manned ambulance, where the on-board anaesthesiologist assumed the role of MSC.

The OUH-HEMS was dispatched 25 minutes after the bomb detonated. The anaesthesiologists and the HEMS paramedics of both OUH-HEMS crews went to the scene via rapid response cars, whereas the pilots shuttled several units of LESS stretcher bags [10] from the HEMS base to the scene by car. Additional personnel from OUH-HEMS were also called in and participated in the on-scene work. Two neighbouring HEMS units (Arendal and Ål) were also dispatched for the incident in Oslo.

A total of 41 ambulance units and four HEMS units were involved in the EMS activities following the Oslo government district bombing. The EMCC of Oslo dispatched and controlled all the prehospital medical services throughout the mission and coordinated the allocation of health assets, in close corporation with the ASC on site.

### Triage and evacuation

**Figure 2:**
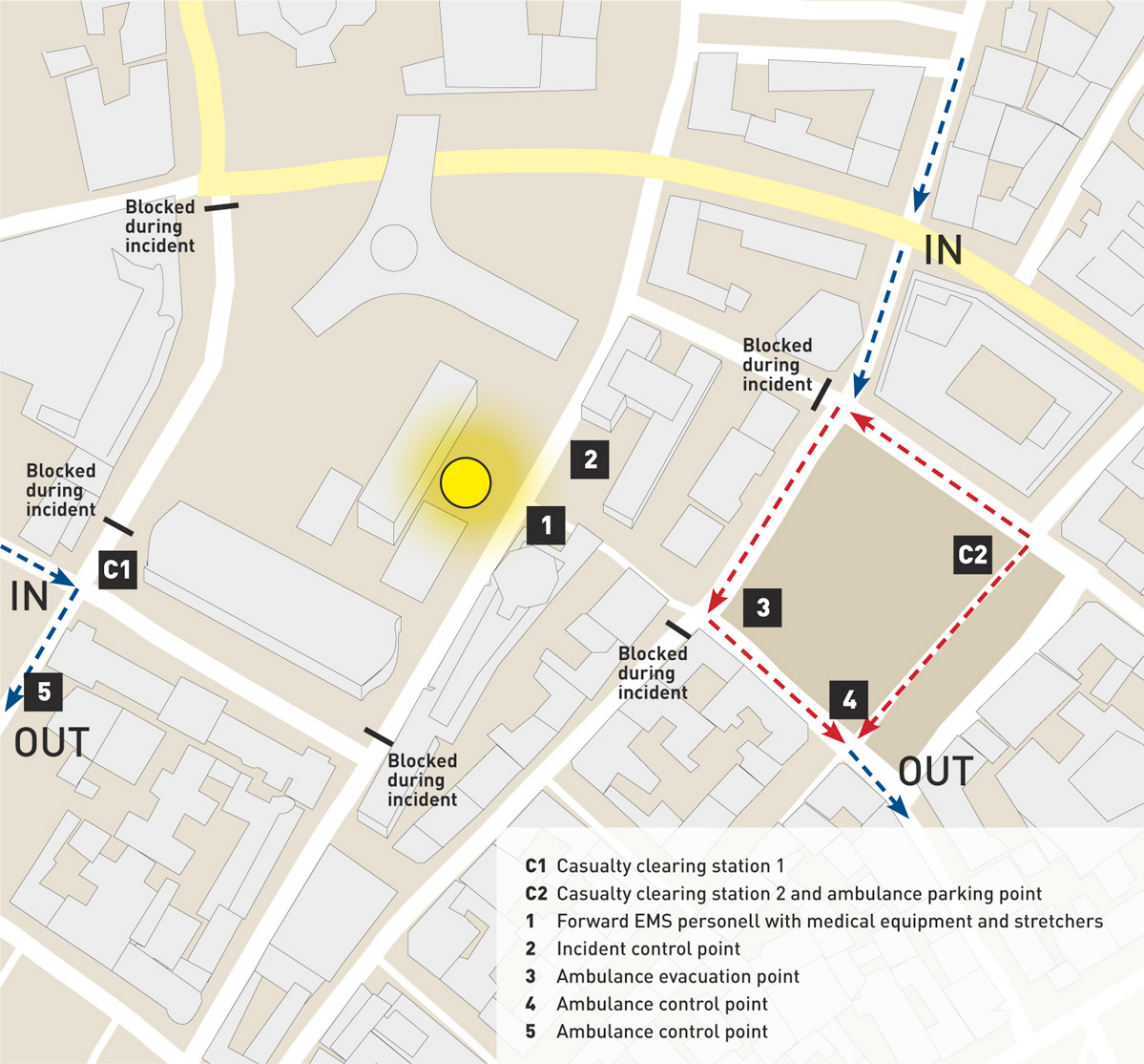
**Map of the Oslo government district depicting the organisation of the EMS response and evacuation routes for ambulances**.

Two casualty-clearing stations (Figure 2) were established because no single evacuation corridor from the scene could be established. Most victims were processed through casualty-clearing station 2 (Figure 2).

The casualty clinic in Oslo received 64 victims from the government district bombing site in the first two hours following the attack. Only one of the victims treated at the casualty clinic was admitted to hospital. In total, 12 trauma victims were transported directly to hospitals in Oslo from the bombing site, and 10 of them were transported to OUH-U. All of the seriously injured victims were transported by ambulance. Casualties suffering from minor injuries were also transported to the casualty clinic by other vehicles, such as police cars, fire department vehicles and a bus requisitioned by the EMS at the bombsite.

At 17:00, the situation was considered to be under control, and a decommissioning of ambulance resources from Oslo was initiated. A heightened preparedness was maintained, however, in case additional victims were found and as a stand-by for the rescue personnel involved.

### The Utøya island scene

Table 3 shows an overview of the events related to the EMS response following the shooting at Utøya island with point of time.

**Table 3:** Timeline of the EMS response to the Utøya island shooting (estimated points of time in italics).

Event	Time
Reports of shooting at Utøya island reaches Buskerud EMCC	17:24

First ambulance units dispatched by Buskerud EMCC	17:24

First EMS unit arrives near Utøya island	17:33

A major incident declared in VVE.	17:45

VVE hospitals Drammen and Ringerike activate their major incident plan (Asker Bærum already activated their major incident plan following the Oslo bombing)	17:45

Utvika quay briefly declared secure by local police	17:55

First victims arrive on the mainland shores east of Utøya island	18:05

First HEMS unit arrives at deployment site south of Utøya island	18:10

Last HEMS unit arrives at deployment site south of Utøya island	18:25

First organised casualty-clearing station established at Utvika quay	18:50

Second casualty-clearing station established at Storøya	19:05

First EMS personnel arrive at Utøya island	*19:40*

First casualty-clearing station closed	*19:45*

First patient from Utøya island arrives at Oslo University Hospital Ullevål	19:57

Last patient from Utøya island arrives at Oslo University Hospital Ullevål	21:30

Last HEMS unit leaves secondary casualty-clearing station	22:20

Second casualty-clearing station closed	*23:00*

EMCC = Emergency Medical Communication Centre, EMS = Emergency Medical Service, HEMS = Helicopter Emergency Medical Service, VVE = Vestre Viken Health Enterprise

### Alarm, dispatch and initial response

The first calls from victims at Utøya island to the EMCC of Buskerud regarding the shooting were received at 17:24. The first ambulance units were dispatched immediately but were held back when they reached the Utøya area because the police had not secured the area. The landside ferry quay of the Utøya ferry (Utvika quay) was briefly declared secure a half hour later, but the arriving ambulance units were soon pulled back again when bullet impacts were observed in the water nearby. The hotel at Sundvollen (Figure 1) was temporarily chosen as the next clearing station for victims arriving from Utøya island.

The first HEMS unit to arrive in the area flew by Utøya island at 18:05 but could not land on the island because of the ongoing shooting. The other HEMS units were routed to a deployment site on the main road south of Utøya island. Because of a low cloud base and fog in the Utøya island area, one HEMS unit deployed by rapid response car directly from the Oslo scene. Their helicopter was used to ferry four additional HEMS physicians from OUH to the deployment site south of Utøya island. In total, three intact HEMS units, six additional HEMS physicians, two nurses and one paramedic from OUH-HEMS and a number of ambulance units were standing by at this deployment site. Two additional HEMS units and two SAR helicopters were still en route to the area. Several local ambulances were already in the area, and more than 20 ambulances and two ambulance buses had been released from OUH. The EMCC of Buskerud dispatched and controlled all the prehospital medical services throughout the Utøya island mission and coordinated the allocation of health assets, in close corporation with the ASCs on site.

### Triage and evacuation

Soon after the shooting started, some of the victims with no injuries or minor injures escaped the attacker at Utøya island by swimming towards the mainland. The first victims to reach the shore arrived scattered over a large area and were attended to by civilians in nearby houses as well as by ambulance personnel and a local GP who by this time were located just above the Utvika quay.

**Figure 3:**
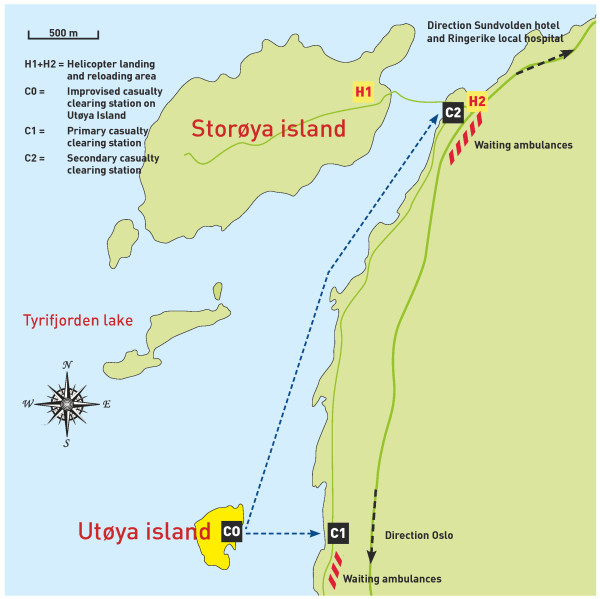
**Map of the Utøya island scene depicting the organisation of the EMS response and evacuation routes**.

The first organised casualty-clearing station was established at Utvika quay when police again declared the area secure (Figure 3). Seven HEMS physicians, two nurses, two local GPs and one anaesthesiologist deployed from VVE engaged in triage on the shores of the Utvika quay area together with ambulance personnel from multiple EMS systems. A local EMT acted as the ASC at this site. Local police secured the area, and local fire and rescue personnel assisted in patient care and rescue. Victims from Utøya island were evacuated on small private boats ferried by local civilians and tourists from a nearby camping site. Most of these victims were physically unhurt but some of them were mildly hypothermic from swimming in the cold waters of Tyrifjorden lake. An estimated number of 10 to 15 of the victims who arrived on these boats in the initial phase had suffered trauma from one or more gunshots. Notably, several of the injured had received crucial first aid from other victims, ferryboat personnel and the police before and during the transport across the lake. Apart from triage for transport, the medical treatment was limited to intravascular access and analgesia during the primary survey. Two critically injured victims were intubated, and one also received thoracic drainage en route to the hospital. All injured victims assessed at Utvika quay were transported directly to the nearest helicopter evacuation point or hospital as soon as possible. In two cases, physicians from the casualty-clearing station accompanied the patient in the ambulance to continue treatment en route to the helicopter evacuation point.

**Figure 4:**
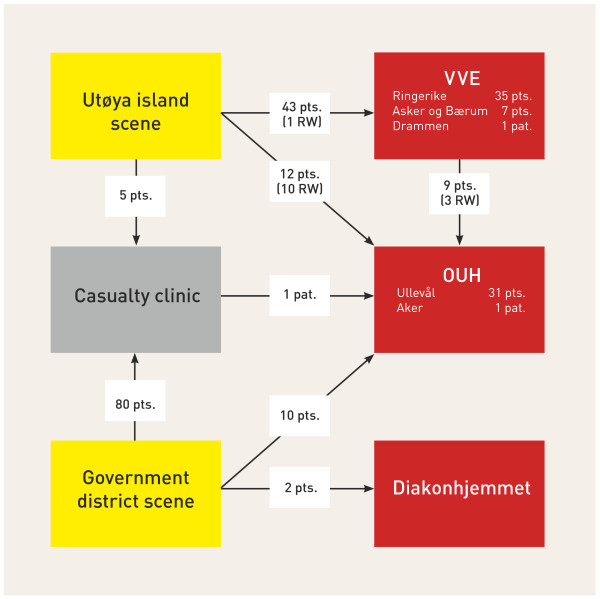
**Patient flow from both scenes to hospitals and institutions that received victims**.

The location of the first casualty-clearing station was close to the scene at Utøya island, but this location proved to be ineffective for the further evacuation of patients. The area was too small and narrow for helicopters to land, and only a small, steep and narrow gravel road connected it to the main road. Ambulances had to drive backwards down the gravel road to pick up patients, and in some cases, patients were carried up to the main road to the waiting ambulances. The main road, a narrow county road, was heavily congested with ambulances, rescue vehicles and private vehicles and made further evacuation difficult. A secondary casualty-clearing station was therefore set up at the bridgehead to Storøya island (Figure 3). This site was chosen because it was a safe distance from the gunshots on Utøya island, which was still unsecured, and because it could accommodate a number of helicopters. One of the OUH-HEMS physicians who arrived at this site with a patient from the Utvika quay acted as the MSC, and this physician worked with the ASC from the local ambulance service to organise triage, primary care and transport for victims arriving directly from Utøya island by boats and from the primary casualty-clearing station at Utvika quay. Seven teams were organised with at least one anaesthesiologist and one assistant in each team. Six HEMS units, two SAR helicopters and 42 ambulances were available for transport. Figure 4 illustrates the patient evacuation routes from Utøya island and from the Oslo government scene.

Approximately 60 flight movements were registered during the Utøya island mission, with a peak of 30 in one hour. Because of bad weather conditions, several flights occurred under Instrument Flight Rules and used the GPS-based instrument approach for the OUH-U helipad.

The primary casualty-clearing station at the Utvika quay area was evacuated again after less than one hour of operation when the car of the perpetrator was discovered in the area. Because the police could not rule out the presence of explosives in the car, all inbound victims were redirected to the secondary casualty-clearing station, and victims already at the first casualty-clearing station were evacuated. By this time, a team of one HEMS physician and three paramedics had set course for Utøya island to establish an advanced casualty-clearing station. The team was initially denied access to the island by the police on the island, but later, they were allowed ashore and were followed by a second team of three HEMS physicians, one physician from VVE, one nurse and two paramedics. The team on the island remained under police protection throughout the evening. Most of the victims passing through this casualty-clearing station were physically unhurt, but four victims with gunshot wounds were managed by the EMS group on Utøya island before evacuation. The team was gradually reduced during the evening to two HEMS physicians and three paramedics. This team participated in a final search for survivors under police protection after midnight, but no survivors were identified. The team remained on the island until 01:30, when an ambulance crew replaced them.

The HEMS units were gradually released from the secondary casualty-clearing station after the last patient was delivered at OUH-U. However, both OUH-HEMS were dispatched for transferring patients from the local hospitals to OUH-U during the night. The next day, four more victims from the Utøya island shooting were transferred by air to other hospitals in Norway: two by helicopter and two by fixed-wing air ambulance.

The secondary casualty-clearing station was closed between 22:30 and 23:00 but remained the base for SAR personnel in the search for deceased victims in the lake and the surrounding area through the night.

All seriously injured victims who were treated and transported from the casualty-clearing stations were alive upon arrival at the hospital. One victim subsequently succumbed to the injuries. Numerous uninjured victims, their relatives and the relatives and friends of the casualties were treated by local physicians and community health care employees who were gathered in the hotel at Sundvolden. This service remained operative for several days.

The perpetrator of the incidents in Oslo and on Utøya island was apprehended on the island by police special forces at 18:33. He did not resist arrest and was captured alive. Throughout the night, it remained unclear whether there were further perpetrators on the island, and the island was first declared safe the next day. The shooting at Utøya island left 69 dead and almost as many physically injured.

## Discussion

### Summary of events

The core of the prehospital efforts directly related to the attacks took place over a period of 10 hours. The time between the contact of the first victim with specialised prehospital health resources and the evacuation of the last victim was approximately one and a half hours in the Oslo incident and two hours in the Utøya island incident. The last victim was transported from the Utøya island incident six hours after the bomb detonated in Oslo. By this time, ground ambulances and HEMS units from eight different health enterprises had been involved in the efforts to treat and transport victims. Both scenes were organised according to the framework for the management, organisation and coordination of mass casualty scenes [9]. With the exception of the casualty-clearing station at Utvika quay, all casualty-clearing stations in both incidents were organised according to this framework. At Utvika quay, the role of the MSC was never formally established. However, because of the relatively large number of physicians with prehospital critical care competence available on the scene, triage appears to have been managed successfully.

### The prehospital challenges of July 22, 2011

#### Geography and EMS systems

Other terrorist attacks in recent years, including the attacks in Istanbul in 2003 [13], Madrid in 2004 [14] and London in 2005 [15], also presented with multiple scenes, which creates a tremendous challenge for the EMS systems. However, in contrast to these attacks, which all occurred in urban areas with short transport distances to hospitals, the scenes of the attacks of July 22, 2011 differed substantially in terms of geography, infrastructure, EMS system and distance to specialised health institutions. The attacks occurred within the catchment areas of two different EMS systems and hospital enterprises. Only the Utøya island scene occurred in an area of overlap between the HEMS of OUH and VVE, and OUH-U is the only hospital that covers both scenes as the regional trauma centre of Southeast Norway. These factors complicated the rescue efforts, but the location of both scenes in the central regions of Norway with a high density of prehospital and hospital resources that were able to cooperate across enterprise and system boundaries, somewhat mitigated these factors. Additionally, at the scene in Oslo, a relatively limited number of victims required specialised care, and the excess EMS and HEMS resources in the region could be reallocated when the Utøya island incident started because the Oslo incident was de-escalating concurrently.

#### Safety of the EMS personnel

EMS personnel in Norway are not required to operate under conditions that can pose a threat to personal safety. If ongoing violence is suspected, EMS personnel are normally allowed to advance only after police declares the scene secured. In some scenarios, EMS personnel can enter an area with dedicated police protection, although the area has not been declared completely secured. There is however little support for the use of EMS personnel in such "*hot zones*"; the evacuation of patients to "*cold zones*" seems more effective [16]. On both scenes on July 22, 2011, EMS units followed police directions. On several occasions, EMS units had to withdraw or evacuate because of security concerns. No EMS units were issued special protective gear, not even the personnel that operated on Utøya island. The use of personal protective gear by EMS personnel, besides uniform and helmet, is not common in such situations. In our opinion, the safety of the EMS personnel involved was cared for, according to standard operating procedures in the EMS systems involved.

#### Triage and evacuation

There is no standard for prehospital triage in Norway [8], and to our knowledge, no single system was used for triage in any of the scenes on July 22, 2011. All victims attended to by the HEMS and EMS were assessed using the implemented principles for primary survey adopted from Advanced Trauma Life Support [17] and Prehospital Trauma Life Support [18]. The tagging of victims was not performed because immediate transport was possible as soon as the victims were evacuated to the nearest casualty-clearing station. During the initial hours of both incidents, large numbers of victims were anticipated. Triage and evacuation plans were formed with large numbers in mind. At the Utøya island scene, a massive evacuation was planned and the use of other regional hospitals was anticipated if OUH-U could not absorb the patient load. It remains unclear whether a unified system for triage in major incidents could have changed the outcome or altered the decisions and plans made on this day. In our opinion, triage and evacuation was successful, since only the most seriously injured were transported to the trauma centre of OUH-U and no victims died before arriving hospital because of delayed transport. We believe that the most important factor that contributed to this seeming success was the competence of the specialised prehospital personnel, with their skills and knowledge of emergency medicine and their knowledge of the EMS system and local hospital structure.

#### Patient flow and communication

Similarly to most incidents of this magnitude, the victims quickly spread over a large area. In the Oslo incident, the rapid control of victims in the outdoor areas was achieved. The greatest challenge was determining how many victims were still in the buildings and how to evacuate them. The new encrypted digital radio system helped to ensure stable radio communication between resources and contributed to maintaining the control of patient flow.

In the Utøya island incident, a large number of victims evacuated themselves to the mainland in the first hour. Local health personnel were spread out in this initial phase, and although a meeting point had been designated at a hotel at Sundvollen, no organised casualty-clearing station was established until approximately 80 minutes after the shooting started. Therefore, complete control of all victims was impossible to obtain in the first phase. The limited coverage and performance of the old analogue emergency radio system in the area contributed to some confusion about the location of the casualty-clearing station and evacuation point at the bridgehead to Storøya island. A few victims who were triaged for direct transport to OUH-U were therefore transported by ambulances to the local hospital, Ringerike. In total, seven victims with severe gunshot injuries arrived at Ringerike Hospital and were successfully received and stabilised.

#### Emergency equipment

The overall impression is that sufficient medical equipment was available at both scenes and that little equipment was actually used because the focus was on rapid triage and evacuation. The LESS stretchers [10] were probably the most useful rescue equipment. At both casualty-clearing stations in the Utøya island incident, LESS stretchers were laid out in the patient arrival/triage zone and stayed with the patients throughout the evacuation, as intended. We believe that this process helped to reduce patient discomfort and improved logistics. The availability of LESS stretchers was limited, however, because they are not available in all EMS systems.

#### HEMS operations

None of the HEMS helicopters were involved in the police operation at any of the scenes, and the police did not request support from the civilian HEMS aviation assets. The light HEMS helicopters were chosen as the primary means of patient transport by air because of their greater efficiency in operation, whereas the larger SAR helicopters were held in stand-by for the transport of large numbers of spontaneously breathing patients if the patient load exceeded the capacity of the six HEMS units. The greatest challenge in the HEMS operation proved to be coordinating the helicopter activity in poor weather conditions, uncontrolled airspace and an unsettled security setting. In addition to the six HEMS helicopters and two SAR helicopters, three additional helicopters from the Royal Norwegian Air Force, one police helicopter and two press helicopters were in the area at different times. Despite these challenges, all HEMS and SAR helicopters were able to communicate and organise adequate improvised landing sites and patterns for landing and take-off near the casualty-clearing station.

### Limitations

The communications log (AMIS) of the EMCCs in Oslo and Akershus failed as the result of overload, and most of the data pertaining to the activities of OUH resources were lost. The data presented in this article are therefore based largely on data from other sources. Accordingly, the time sequences presented are not entirely reliable. We believe, however, that our reconstruction is fairly accurate.

The recordings of the patient flow in the initial phase of the evacuation from both scenes were not complete. In hindsight, all victims were accounted for, but in some cases, the evacuation points from which specific individuals were evacuated are still unclear. A reconstruction of these events would probably have been possible from interviews with the victims, the EMS personnel and the police. However, such an extensive data collection process was not within the scope of this descriptive study.

## Conclusion

The terrorist attacks in Norway on July 22, 2011 elicited a massive prehospital response involving units from eight different health enterprises. Despite the occurrence of two scenes within a short time span and with a significant geographical distance between them, a large number of EMS and HEMS resources from different systems could be activated and utilised. The time to treatment was delayed for many victims at the Utøya island because of safety concerns and geographical challenges. However, we believe that the EMS response was successful under the given conditions. The lack of a robust radio communication system at the Utøya island scene and the breakdown of the communications log (AMIS) are issues that need to be addressed. We also believe that the experiences warrant a "common language" in the management of major incidents, perhaps in the form of a national standard major incident triage.

## List of abbreviations used

AMIS: Acute Medical Information System; ASC: Ambulance Scene Commander; EMS: Emergency Medical Service; EMCC: Emergency Medical Communication Centre; EMT: Emergency Medical Technician; HEMS: Helicopter Emergency Medical Service; LESS: Light Emergency Stretcher Systems; MSC: Medical Scene Commander; OUH: Oslo University Hospital; SAR: Search and Rescue; VVE: Vestre Viken Health Enterprise.

## Competing interests

The authors declare that they have no competing interests.

## Authors' contributions

SJMS drafted the manuscript and coordinated the writing process and data presentation. RR helped draft the manuscript and contributed to the data collection from the Oslo scene. MR helped draft the manuscript and develop the figures. ANR helped draft the manuscript and develop the figures. AET helped draft the manuscript and contributed to the data collection from the Utøya scene. TS, HJH and JEN helped draft the manuscript. MS helped draft the manuscript and coordinated the data collection. The Collaborating group helped verify the data collected. All authors read and approved the final manuscript.
